# Editorial: Mental illness, culture, and society: Dealing with the COVID-19 pandemic

**DOI:** 10.3389/fpsyt.2022.1073768

**Published:** 2022-11-04

**Authors:** Renato de Filippis, Samer El Hayek, Mohammadreza Shalbafan

**Affiliations:** ^1^Psychiatry Unit, Department of Health Sciences, University Magna Graecia of Catanzaro, Catanzaro, Italy; ^2^Medical Department, Erada Center for Treatment and Rehab, Dubai, United Arab Emirates; ^3^Mental Health Research Center, Psychosocial Health Research Institute, Department of Psychiatry, School of Medicine, Iran University of Medical Sciences, Tehran, Iran

**Keywords:** coronavirus, general population, lockdown, healthcare workers, mental health, psychiatry, social distancing, vulnerable populations

Since 2020, the spread of the coronavirus and its subsequent clinical manifestation, the Coronavirus Disease (COVID-19) pandemic, have represented a historic event of global significance. The pandemic has affected the quality of life and mental health status of both the general population and patients with mental health problems (1–3). In the beginning, the health and social consequences were mainly linked to the direct life-threatening risk posed by an unknown and highly fatal respiratory disease, for which no treatment or vaccine were available (4). Therefore, disease prevention, through physical distancing of the general population and isolation of cases, was considered the most effective measure to minimize the spread of the virus (5).

Lockdown and isolation measures have, therefore, characterized much of the global fight against COVID-19. These measures unfortunately had serious economic, educational, social, and mental health repercussions for individuals and societies (6). While the effects of lockdown and isolation have been extensively studied in individuals with mental health problems, less has been explored on the role of socio-cultural, environmental, and local factors in facing COVID-19 (7). Therefore, in this Research Topic, we look at the effects of the pandemic on mental health from the lens of local and sociocultural factors. We particularly look at three groups of individuals: the general population, special vulnerable populations, and health care providers.

In the first section of this editorial, we will provide an overview of the main findings of the publications in our Research Topic assessing mental health outcomes and psychological issues among the general population, amid different waves of the COVID-19 pandemic and from a various range of cultures and societies.

Post Traumatic Stress Disorder (PTSD) has been one of the most studied psychiatric disorders during the outbreak. Shen et al. reported on the prevalence of PTSD and its related factors among the Chinese population, 1 year after the start of the pandemic. The authors used the PTSD Checklist for DSM-5 (PCL-5) among a sample of 2,361 Chinese residents and found a PTSD prevalence of 9.28%. Rajkumar presented an analysis of factors contributing to PTSD amid the pandemic, by analyzing data from 35 countries. He found a positive relationship between PTSD and the COVID-19 case-fatality ratio and power distance. He also noted a trend toward a negative quadratic association between internet usage and PTSD. Lastly, he did not detect significant cross-national effects for government restrictiveness (Rajkumar).

Other mental issues such as mental wellbeing, psychological distress, and coping behaviors were topics further investigated by several studies in our Research Topic. Wong et al. looked at the impact of containment during the pandemic and coping behaviors. The authors reported that the influence of containment on individual psychological aspects was prominent, followed by impact on wellbeing and lifestyle. Furthermore, physical coping strategies and mindfulness were most commonly reported (Wong et al.). Alghamdi et al. investigated psychological distress using the Depression, Anxiety, and Stress Scale-21 (DASS-21) among 2,252 participants of the general population of Saudi Arabia. They found the DASS-21 mean score of participants to be within normal range, with the mean score of healthcare workers significantly higher than that of other participants. Age, gender, and history of contact with confirmed COVID-19 cases were significantly associated with higher DASS-21 scores (Alghamdi et al.). Babicki et al. performed a nation-wide study on 5,790 Polish individuals using the Beck Depression Inventory (BDI), the Generalized Anxiety Disorder Assessment (GAD-7), and the Manchester Short Assessment of Quality of Life (MANSA) in the first three waves of the pandemic. The authors concluded that, as the COVID-19 pandemic progressed, depressive and anxiety symptoms increased. In addition, women, single individuals, and those with prior psychiatric treatment were the most vulnerable (Babicki et al.). In another study, Hu et al. investigated COVID-19 related stress and mental health outcomes among 771 Chinese individuals using an online survey. They reported that resilience mediates the effects of COVID-19 related stress on depression and post-traumatic growth. On the other hand, social support mediates the impacts of COVID-19 related stress on post-traumatic growth, depression, and anxiety (Hu et al.). Menculini et al. performed a two-year observational study on youths in Umbria, central Italy, to assess psychopathological distress amid the pandemic. The authors found anxiety disorders to be the most prevalent. The most frequently used treatment approach was digital mental health services, and psychopharmacological treatment was more commonly provided among the general population (Menculini et al.). In addition, Yong and Zhang used the 12-item General Health Questionnaire (GHQ-12) to evaluate COVID-19 worry and mental health among 1,584 economically active Chinese participants. Almost half (42%) of participants reported being “very worried” or “extremely worried” about the pandemic. This worry was associated with male gender, young age (16–45 years), being unemployed, and having mental health problems (Yong and Zhang). Jang et al. also investigated the relationship between economic loss and anxiety among 911 Korean individuals at two times: during the early months of the pandemic and 6 months later. The authors concluded that, in the early stages of the pandemic, gratitude and perceived stress had moderating effects on this relationship. However, after 6 months, only perceived stress had a significant moderating effect (Jang et al.). Schabus et al. investigated psychosocial burden, risk-perception, and attitudes among 3,848 Austrian individuals from the general population. They found that isolation from family and friends, homeschooling, and economic consequences were perceived as the most stressful factors. They also noted that, compared to non-regular users, regular public media users significantly overestimated hospitalization risk secondary to COVID-19 (Schabus et al.). Sadegh-Zadeh et al. assessed the effects of the pandemic on components of social and mental health using machine learning among a general sample from the United States. They concluded that individuals with previous diagnosis of any psychiatric disorders were most affected by the constraints implemented during the pandemic (Sadegh-Zadeh et al.).

One of the included papers in our Research Topic assessed the correlation between COVID-19 and schizophrenia. In this case-control study conducted in Indonesia, Amin et al. found that the coronavirus infection was more frequent in the schizophrenia group, particularly among older adults.

A few papers investigated social concepts amid the pandemic. In their Ecuador-United States based study, Franklin et al. analyzed overconsumption behaviors during the pandemic. The authors concluded that health consciousness is responsible for stimulating overconsumption behaviors (Franklin et al.). Pratt and Carr published an opinion piece about the effects of the pandemic on the Japanese society, more specifically tackling the postponement of the Olympic games. Last but not the least, in a qualitative study among school-based professionals in Appalachia, Haliwa et al. reported about the overall positive attitude of participants toward mindfulness training.

To summarize, this first part of our Research Topic highlights and emphasizes the importance of mental health among the general population during the COVID-19 pandemic. The included papers suggest different practical approaches to improve the mental wellbeing of societies, through the help of policymakers and national goverments.

In the second section of this editorial, we will discuss studies in our Research Topic that looked at how the COVID-19 pandemic affected the mental health of special populations. In particular, the studies focused on students, veterans, teachers, pregnant women, immigrants, older adults, and patients with severe medical comorbidities, providing data otherwise largely missing in current literature panorama (8). This part of our Research Topic encompassed a total of 19 papers: 13 original research articles, three reviews, one brief research report, one perspective paper, and one opinion piece, thus offering a wide panorama of study designs and formats covering most aspects related to the implications of COVID-19 on the mental wellbeing of vulnerable populations.

The large percentage of articles with original data demonstrates the flourishing and recent international scientific production around COVID-19, with data often collected through surveys or telemedicine, which have been adapted due to the impossibility of physical contact during lockdown measures (9).

The paper by Cerami et al. defined the clinical framework of reference when talking about fragile subjects at greater risk of developing serious consequences secondary to COVID-19. Through an online survey distributed among 1,258 residents during the first pandemic wave and the consequent first general lockdown in Italy, the authors highlighted the importance of social vulnerability to environmental stressors, such as social distancing, isolation, and loneliness, to explain the individual perception of the impact of COVID-19 emergency on health, beyond physical frailty (Cerami et al.). The authors concluded that the early identification of individuals most exposed to the social consequences of COVID-19 could direct governments to allocate more resources and plan strategies to contain consequences, and, in the case of this Research Topic, to phenotype these vulnerable categories to better focus research on them.

Following the same path, Kumar et al. conducted a rapid review to investigate the trends in psychological impacts, coping ways, and public support during the COVID-19 pandemic in the United States. They reported results from 35 included studies, mainly involving vulnerable individuals, suggesting that women, children, elderly, and racial minorities have been affected by a lack of adequate support for psychological wellbeing during the crisis.

Alternatively, hospitalization, quarantine, and social isolation were negative prognostic factors for the mental health of patients testing positive for COVID-19 (10). In the cross-sectional survey conducted by Ouanes et al., the authors evaluated the physical and psychological wellbeing of 141 inpatients with COVID-19, 99 quarantined patients, and 285 healthy controls. They found better psychological growth and enhanced resilience in patients with social support from family and friends, and easy access to mental health screening and care, highlighting the importance of the socio-cultural context for the support of the most fragile patients.

Women's health was also severely affected by the coronavirus outbreak, including the menstrual cycle, pregnancy, and peripartum period (11). Three papers included in this Research Topic dealt with pregnancy and peripartum conditions. Arzamani et al. conducted a review exploring psychological problems (e.g., fear, anxiety, depression) experienced by pregnant women during the outbreak. Their findings pinpoint that mental health issues linked to the pandemic may reduce compliance to effective preventive behaviors in pregnant women, provide unhealthy coping mechanisms, cause inadequate care during childbirth, and have negative effects on the prognosis of pregnancy and fetal development. Similar results were found in the study carried out in Italy by Orsolini et al., regarding perinatal depression caused by fear and anxiety related to COVID-19. This study was among the first to investigate, in detail, which COVID-19-related psychopathological determinants may predispose to perinatal depression. The authors concluded that isolation, quarantine, lockdown, and deprivation of normal social support led a total sample of 184 perinatal outpatients to have increased levels of anxiety, fear, and psychological distress, independently of their previous psychiatric history (Orsolini et al.). Finally, the study conducted by Ma et al. confirmed previous results as it assessed the psychological impact of the COVID-19 pandemic among pregnant women in mainland China. This study, carried out as a cross-sectional survey enrolling a large sample of 1,078 participants, stated that despite increased family and social support, more than half of enrolled pregnant women reported increased feelings of being horrified, apprehensive, and helpless secondary to the pandemic.

Similar results can be found among children and elderly, who are considered as the two age groups at greatest risk of suffering from social and relational restrictions. Therefore, in her *wake-up call*, Solerdelcoll outlined the current global interest in the impact of the COVID-19 pandemic on children's mental health, mainly based on speculation, media coverage, and academic studies. In this opinion piece, she pointed out the attention about the need to deepen knowledge and raise awareness of the key cultural and contextual factors affecting children, sketching out the main points on which to act immediately. Similarly, older adults are considered vulnerable individuals who should be protected from the direct and indirect effects of COVID-19 on the general and psychological health. In contrast to many literature results, the group led by López has shown how psychological wellbeing, structured on personal growth and purpose in life, acted as a strong protective factor for 192 people over 60 years old during all the pandemic phases in Spain.

Several articles delved into the effects of the pandemic on school and education. During the early stages of the pandemic, one study carried out among college students in China investigated the effect of perceived threat avoidability of COVID-19 on coping strategies and anxiety (Wu et al.). The authors found that the perceived threat of coronavirus infection exacerbated anxiety symptoms in students. These symptoms were only partially mitigated by coping strategies. These findings were confirmed by a rapid review of the literature examining the COVID-19 influence on five aspects of mental health: emotional features, personality, interpersonal relationships, learning behavior, and employment options among undergraduate students (Shi et al.). Teachers also suffered from the lockdown measures which tremendously affected school systems and educational problems (12). In an original study settled in Bangladesh, Hossain et al. found a high prevalence of depression, anxiety, and stress during the second wave of the COVID-19 pandemic among teachers, especially those who were males and older.

Another large population considered at risk for the consequences of COVID-19 are patients with severe physical comorbidities. In this context, we collected three articles that respectively evaluated patients living with type 2 diabetes mellitus (T2DM) (Sayed Ahmed et al.), dementia (Mohammadian et al.), and immunocompromised health (Heesen et al.). The high prevalence of both T2DM and coronavirus infection around the world makes the overlap between these two diseases not only very likely but also extremely common (13). Therefore, it does not come as a surprise that living with T2DM during the COVID-19 pandemic was linked with increased distress, depression, and anxiety symptoms in Egypt (Sayed Ahmed et al.). Similarly, dementia and cognitive decline seem to be negative prognostic factors in individuals infected with COVID-19. In this regard, Mohammadian et al. found a direct relationship between cognitive decline and the psychological impact of COVID-19 in both patients and their caregivers in Iran. Lastly, the reduction of the immune defenses of the body represents an important risk factor for the development of infectious diseases, including COVID-19. In this sense, Heesen et al. studied the participation of immunocompromised patients in Germany in social activities, before and after completing the vaccination cycle. He concluded that vaccination returns to special populations a good level of social interaction that was lost with physical isolation (Heesen et al.).

The study by Kilic et al. evaluated infection risk and vaccine status in patients with attention deficit and hyperactivity disorder (ADHD). The authors found that the COVID-19 vaccine is acceptable and receiving the vaccine is typically endorsed by patients with ADHD. In addition, being diagnosed with ADHD did not provoke any kind of mental disturbance in the sense of perception of danger from COVID-19 (Kilic et al.). However, despite the growing evidence on the effectiveness of vaccines, vaccination hesitancy remains a widespread phenomenon around the world. In some areas, including Latin America and the Caribbean, this phenomenon appears particularly marked; we have therefore included a perspective article in our Research Topic that particularly tackles this subject (Faria et al.).

Lastly, this section of our Research Topic included two articles assessing the impact of the pandemic on two other vulnerable populations: veterans and immigrants. Veterans are already at a high risk to develop anxiety, sleep disorders, depression, and PTSD (14). Therefore, assessing how the COVID-19 pandemic might have impacted their mental health is critical. According to Stellman et al., previous military experiences affected coping with COVID-19 both positively and negatively, and may have helped instill useful personal health behaviors in veterans. When it comes to immigrants, we included an illustrative work on how migrants coped with the COVID-19 pandemic, with a peculiar study about the experience of Afghan immigrants in Iran (Mohammadsadeghi et al.). COVID-19 and the subsequent lockdown and isolation measures caused further trauma, adding to the effects of previous experiences of war and migration, with the consequent appearance of fear of losing control, being overwhelmed, and inability to cope (Mohammadsadeghi et al.).

Lastly, one important aspect to evaluate is the effect of working place infection control practices on workers' psychological distress. In this line of thought, Kodama et al. found that some infection control practices reduced workers' distress while others worsen it. Therefore, employers need to consider, not only infection control practices, but also the worsening mental state of employees following a decrease in income caused by such measures (Kodama et al.).

One notable aspect of the current impact of the COVID-19 pandemic on mental health was how the pandemic affected individuals working within the medical field, while particularly noting the intertwining roles of culture and society (15).

Accumulating evidence indicates that the COVID-19 pandemic and associated public health crises have had a disproportionately negative impact on healthcare workers (HCWs) (16). Due to the high levels of psychological stress, this group has been experiencing worsening mental health outcomes. These psychological problems, affecting physicians, nurses, and other HCWs, include depression, anxiety, insomnia, and PTSD (17, 18). Therefore, this last section of the editorial is particularly dedicated to studies in our Research Topic looking at the impact of COVID-19 on healthcare professionals (HCPs).

Several articles assessed the prevalence of mental health symptoms and disorders among HCWs. The article by Almalki et al. looked at the prevalence of depression, anxiety, and stress among physicians, pharmacists, nurses, and other HCWs in Saudi Arabia using the DASS-21. Among 501 HCWs, the estimated prevalence rates of depression, anxiety, and stress were 54.69%, 60.88%, and 41.92%, respectively. HCWs with chronic diseases, nurses, and HCWs from the southern region of the country were more likely to suffer from depression and stress. Further, individuals with positive COVID-19 test results showed a greater proportion of depressive symptoms compared to others. In addition, knowing someone who died due to COVID-19 and having a chronic illness were predisposing factors for anxiety (Almalki et al.).

Along the same lines, Hajebi et al. looked at the mental health of HCWs in Iran, albeit using the Patient Health Questionnaire-9 (PHQ-9), Generalized Anxiety Disorder-7 (GAD-7), and Copenhagen Burnout Inventory, and found comparable results. Half of the participants (53%) either had a generalized anxiety disorder or major depressive disorder or both disorders. Moderate and high levels of burnout were seen among 48.9% of the study participants. The prevalence of mental disorders and burnout was significantly higher among females and those working in hospitals compared to primary healthcare centers. Predictors of mental disorder and burnout were “*worry about children and old members of the family*,” “*family worries for my health condition*”, and “*lack of specific effective treatment for COVID-19*” (Hajebi et al.).

The influence of COVID-19 among occupational and physical therapists in Kuwait was particularly assessed in the paper by Alnaser et al. This cross-sectional study included 98 participants and examined self-reported anxiety (*via* the GAD-7) and somatic symptoms (*via* the modified PHQ, mPHQ-15). The authors found that 14%, 38%, and 21% of participants had mild, moderate, and severe anxiety, respectively. In terms of somatic symptoms, 20%, 38%, and 29% of participants reported mild, moderate, and severe symptoms, respectively. GAD-7 and mPHQ-15 scores were moderately positively correlated. The therapists perceived that the quality (76%) and effectiveness (20%) of their rehabilitation services were negatively affected by the pandemic (Alnaser et al.).

In their qualitative study, Rouhbakhsh et al. looked at themes about the perception of stress among HCPs during the pandemic. Twenty HCPs were recruited from a teaching hospital in Iran and included physicians, nurses, and other paramedics. Participants reported a wide range of psychological reactions including anxiety, feelings of guilt, depression, and anger. Uncertainty accompanied by the pandemic and shortcomings in preparation for crisis management were recognized as the two main sources of stress (Rouhbakhsh et al.). Nohesara et al. also carried out qualitative research to study the grief experiences of 12 intensive care unit staff members who experienced the loss of a family member during the pandemic in Iran. The authors found five common themes in the experiences of participants: complex grieving process, new experiences for coping with loss, more empathy for patients, change in the meaning of death, and the need for support in workplaces (Nohesara et al.).

Shifting perspectives, Chen et al. looked at anxiety and depression states among 428 dry eye patients in China. Patients were tested with the Ocular Surface Disease Index, Short Healthy Anxiety Inventory, Hospital Anxiety and Depression Scale, and Pittsburgh Sleep Quality Index. The incidence rates of depression and anxiety were 26.87 and 27.34%, respectively. One-quarter of participants (24.30%) had comorbid anxiety and depression. Higher education levels, a shorter course of the disease, lower health anxiety levels, and better subjective sleep quality were significantly associated with reduced depressive and anxiety symptoms among patients (Chen et al.). The study by Lan et al. also evaluated sleep disorders related to COVID-19. The authors suggested that an individual's perceived COVID-19 crisis strength indirectly affects their life satisfaction and sleep quality, *via* their perceived risk of being infected (Lan et al.).

Besides the above clinical reports, Zhou et al. and validated a machine learning-based model to predict depression symptoms among HCWs during the pandemic. The model was created using survey data collected from 2,574 HCWs in hospitals designated to care for COVID-19 patients in China. The machine learning models highly consistently identified and ranked risk predictors for depression. Self-perceived health status factors always occupied the top five most important predictors. Other top predictors were worries about infection, working on the frontline, a very high level of uncertainty, and having COVID-19-like symptoms. The authors concluded that the application of such machine learning models could support decision-making on the implementation of mental health interventions to support HCWs (Zhou et al.).

The last paper by Halms et al. presents a scoping review and evaluation of guidelines and recommendations published for the support of HCWs during the pandemic. The study included 41 articles published between April 2020 and May 2021. The authors clustered the retrieved guidelines and recommendations into four main categories: social/structural support, work environment, communication/information, and mental health support. Although there was substantial agreement across the recommendations, empirical evidence on their effectiveness was lacking. More importantly, most recommendations were developed without involving HCWs or related stakeholders (Halms et al.).

Taken together, this section covers the effects of COVID-19 on HCPs and updates readers on the latest research in this field. We hope that this work will encourage researchers to further explore the relationship between the COVID-19 pandemic, mental health, and HCPs. We also hope that it will provide insights into how to support HCWs appropriately and effectively during this era.

In conclusion, all papers included in the Research Topic and described in this Editorial piece focus on the mental health status of the general population, vulnerable populations, and HCPs during several phases of the COVID-19 pandemic. Considering the novelty and paucity of evidence available about the consequences of lockdown measures and physical distancing on various groups and within different sociocultural backgrounds, the articles collected in this Research Topic shed some light on the mental health implications of the pandemic throughout a wide range of settings.

Although research on COVID-19 and mental health has already produced a large amount of data on many aspects, we believe that the clinical framework offered in these articles provides a different and original point of view that could lead to more targeted and specific use of forces and resources, which may interest clinicians and researchers all over the world.

## Author contributions

All authors listed have made a substantial, direct, and intellectual contribution to the work and approved it for publication.

## Conflict of interest

The authors declare that the research was conducted in the absence of any commercial or financial relationships that could be construed as a potential conflict of interest.

## Publisher's note

All claims expressed in this article are solely those of the authors and do not necessarily represent those of their affiliated organizations, or those of the publisher, the editors and the reviewers. Any product that may be evaluated in this article, or claim that may be made by its manufacturer, is not guaranteed or endorsed by the publisher.
